# Are virtual anime endorsers a new type of endorser? Examining product involvement as a moderating role

**DOI:** 10.3389/fpsyg.2022.779519

**Published:** 2022-08-30

**Authors:** Huai-Liang Liang, Feng-Hua Yang

**Affiliations:** ^1^Executive Master of Business Administration Program, Dayeh University, Changhua, Taiwan; ^2^Department of International Business, Dayeh University, Changhua, Taiwan

**Keywords:** virtual anime endorser, product involvement, brand attitude, elaboration likelihood model, endorser credibility

## Abstract

Virtual anime endorsement has been prevalent as an advertising strategy, and many companies invest massive amounts of money into virtual endorsements. While previous studies have found that endorser-product congruence is related to consumer brand attitude and purchase intention, it is not known whether moderate incongruence between a virtual anime endorser and a product has a positive influence on brand attitude and purchase intention. This study developed a 1 × 2 experiment to investigate the influences of virtual anime endorser–product congruence and moderate the endorser–product incongruence on consumer brand attitude and purchase intention. Product involvement played a key moderating role in the relationships of virtual anime endorser–product congruence and the endorser–product incongruence with consumer attitudes. A total of 919 participants were recruited from animation-related venues and stores in Taiwan. The findings of this study validated the interaction effects of virtual anime endorser-product congruence and incongruence on these two consumer responses, i.e., brand attitude and purchase intention. This study further investigated the moderating effect of product involvement in the relationships of virtual anime endorser–product congruence and moderate the endorser–product incongruence with consumer responses. The findings of this study provide a valuable reference regarding endorsers and product patterns through which enterprises can maximize their value.

## Introduction

Many marketing studies have focused on endorser–product congruence and the match-up hypothesis (Albert et al., 2017; Liang and Lin, 2018; Schouten et al., 2020). Most companies rely on suitable endorsers to promote their products and invest considerable capital to gain endorsements from celebrities in various public places and the media. Prior studies indicate that many companies have forgone celebrity endorsers in favor of using “virtual anime endorsers” to promote and market their brands and products (Tamaki, 2019; Lin et al., 2021). For instance, the company of the 2020 Tokyo Olympics selected the anime character Son Goku as the ambassador of this event. Jin and Bolebruch (2009) reported that virtual endorsers can enhance consumers’ perceived value and positive brand attitude toward a company’s product offerings. Anime products have experienced considerable growth and increasing profits through social media platforms, such as YouTube. In 2020, the gross market size of anime-related products was USD 24.80 billion (Market Analysis Report, 2021). In addition, Statista Research Department (2021) declared that the Japanese animation industry had reached a revenue of almost 1.24 trillion Japanese yen in 2020. Therefore, the use of virtual anime endorsers in corporate image roles may become a more popular alternative for companies than the use of celebrity endorsers. The animation industry has become an economic lifeline that cannot be overlooked.

Virtual anime endorsers, such as Ronald McDonald, are created using computer-generated graphics and have already replaced some celebrity endorsers (Hsu et al., 2018) and are developed by virtual anime characters and cartoon-like endorsers, such as the Mermaid and Michelin Man. Virtual anime endorsers attract consumer interest and increase purchase intention even though they may not match perfectly with the characteristics of the endorsed product (Aggarwa and McGill, 2007). Endorsements based on an endorser’s attractiveness or personal character are only persuasive for certain products (Schouten et al., 2020). With the popularity of online advertising and marketing, the role of electronically created virtual endorsers is gradually expanding on social media, and their influence on marketing is growing (Hsu et al., 2018). The imagery of these representative characters is skillfully used by companies and corporate brands as a vital method of transmitting messages (Keng et al., 2011). Because virtual characters never undergo aging or changes in appearance, these characters can forever occupy a space in the hearts and minds of consumers and have the capacity to attract the attention of numerous age groups (Hsu et al., 2018). Holzwarth et al. (2006) stated that virtual characters increase customer satisfaction with the retailer, product attitude, and purchase intention. However, few studies have focused on the manner in which virtual anime endorsers endorse products and influence consumer purchasing behaviors and attitudes toward a brand. Virtual endorsers may not be completely beneficial for companies and brand users; therefore, the aforementioned knowledge gaps and their practical implications warrant research.

To judge endorsers and product information, consumers consider the match-up between products and endorsers (Hsu et al., 2018). According to the elaboration likelihood model (ELM; Petty and Cacioppo, 1984), consumers with high levels of product involvement carefully judge product information in relation to product quality. Conversely, those with low-level product involvement are often influenced by outside factors during information processing. Specifically, when congruence is achieved between the appearance of an endorser and an endorsed product, consumers with low levels of product involvement are still easily affected by peripheral clues (e.g., the attraction of source); however, those with a high level of product involvement are unaffected by peripheral clues of no relevance to the product quality. Therefore, this research conducted a literature review within the framework of the ELM in order to provide valuable insight into the aforementioned topic.

This study had three main goals: (1) to examine the interaction effects of virtual endorser–product congruence and virtual endorser–product incongruence on consumers’ purchase intention and attitude toward a brand, (2) to examine the moderating role of the cognitive condition of consumers’ product involvement in the relationships of virtual endorser–product congruence and virtual endorser–product incongruence with consumers’ attitudes toward a brand, and (3) to contribute to the literature on endorser–product endorsement by identifying its main contributing factors. This study may enhance the understanding of endorsement fields.

## Theoretical background and hypotheses

### Elaboration likelihood model

According to the ELM (Petty et al., 1981), consumer ideas about goods are influenced by various external and internal factors. External factors alter consumers’ opinions through central and peripheral channels, whereas internal factors refer to the degree of an individual’s deliberation, during which consumers consider subjective ideas and experiences when interpreting messages as part of their thought processes. Consumers exhibiting a high degree of scrutiny are cautiously review the content of information; therefore, the content of a message is the central processing route. On the contrary, consumers who choose the peripheral processing route and who have a low degree of scrutiny often lack the motivation and ability for thoughtful consideration (Tang et al., 2012).

In the ELM, the central route refers to consumers’ attitudes toward products that are easily affected by product information. This route emphasizes the processing of persuasive messages in a rational and objective manner as well as the investment of individuals into the content of messages (Lee and Koo, 2016). Consumers’ cognitive process or their evaluation of products is primarily a result of their ideas. Liu et al. (2007) report that consumers’ attitudes are informed by the relevant information disclosed about a product, such as its practical suitability. In addition, when the peripheral route is used by consumers who lack a buying motive or when their purchasing power is not sufficiently strong to elicit evaluative ability, attitude changes may be achieved through the peripheral route. These consumers do pay attention to the product information but rather focus on numerous other factors that satisfy their present cognitive understanding. Consumers who adopt the peripheral route do not wish to spend much time or energy analyzing product information and are thus more easily convinced by surface features (Tang et al., 2012).

### Celebrity endorser and virtual anime endorser

Products endorsed by a celebrity endorser may generate the most profit and advertising effectiveness for the company (Till and Busler, 2000; Lee and Koo, 2016). Prior research reported that consistency between an endorser’s celebrity image and the nature of the endorsed product affects consumers’ purchase intention (Liang et al., 2011). The higher a celebrity endorser’s profile, the more they are received and trusted by the audience, which in turn affects the audience’s buying attitude (Sheu, 2010). Therefore, companies may create maximum advertising effectiveness by selecting a celebrity endorser related to the company’s products within the confines of their budget (Bower and Landreth, 2001; Koernig and Boyd, 2009). Corporations spend astronomical capital on endorsement fees to acquire celebrity endorsers and prime-time media advertisements. If an endorser’s moral fiber is considered lacking, the endorsed company’s reputation may be damaged, which may adversely affect its product sales and corporate earnings (Zhou and Whitla, 2013). Unlike celebrity endorsers and movie stars, virtual endorsers rarely display negative publicity (e.g., scandals) or engage in negative behaviors (e.g., crimes; Garretson and Niedrich, 2004). Thus, the use of virtual endorsers to endorse products or brands is increasing (Hsu et al., 2018).

Virtual anime endorsers, such as Ronald McDonald and the Poppin’ Fresh Pillsbury Doughboy, are created using computer-generated graphics and have already replaced some celebrity endorsers and become popular media figures (Hosany et al., 2013; Hsu et al., 2018). A growing number of companies have developed virtual anime characters and cartoon-like endorsers, such as One Piece (manga and anime), the Mermaid, and Michelin Man. These endorser characters have created strong company, brand identification, and remind brand impressions of community. Prior studies have indicated that a virtual or an anime character acting as an endorser is attractive to consumers and strengthens product word-of-mouth and brand image (Hsu et al., 2018; Schouten et al., 2020). When company characters are recognizable, virtual endorsers draw the attention of potential consumers and create strong brand identification. Thus, virtual anime endorsers are increasingly used by numerous international brands, in advertisements, and at physical events. For instance, the 2020 Tokyo Olympics committee selected Son Goku as its ambassador (Tamaki, 2019). Modern advertisers also use virtual characters as alternatives to celebrity endorsers because private behaviors need not be managed in the case of these characters (Till and Busler, 2000; Hsu et al., 2018).

### Credibility effect of virtual anime endorser–product congruence and consumer attitudes

Although theorical research and empirical phenomena have revealed the effectiveness of celebrity endorsements, several factors may alter the effectiveness of such endorsements on consumer’s attitude toward a brand and purchase intentions. First, an incongruence between a celebrity endorser and the endorsed product may negatively affect the effectiveness of the endorsement. Similarly, Schouten et al. (2020) indicated that congruence between a celebrity endorser and product leads to favorable attitude toward a brand and high purchase intentions toward the product. Second, an anime or virtual character endorser rarely occurs scandals and crimes that can adversely affect consumers’ attitude toward a brand and purchase intentions. Zhou et al. (2021) indicated that the characteristics of anime endorsers positively induce advertisement effects. However, celebrity endorsers may behave negatively in public or be marred by negative gossip, which results in less favorable attitudes toward brands and decreased purchase intentions. Therefore, the effectiveness of source credibility in endorsement may be associated with the congruence between an endorser and a product.

Product promotion using endorsers as a marketing method can promote product exposure and increase consumer purchase intention (Fink et al., 2004; Schouten et al., 2020). MacKenzie and Spreng (1992) demonstrated that consumers’ attitude toward a brand and ideas about a product affect their purchase behavior. Attitude toward a brand is a factor used by consumers to evaluate products. Lutz et al. (1983) identified that three attitudes influence consumer purchase behavior: cognitive, affective, and constitutive attitudes. Cognitive factor refers to the knowledge and perception that consumers have about products, and affective attitude indicates consumers’ emotional responses to a product. When consumers are confronted with various products, they may analyze a wide variety of information, which leads to them forming positive or negative opinions about various brands. Prior studies have reported that the majority of consumer purchasing behavior is attributable to consumers’ cognition toward a virtual character and endorsers because these factors affect consumers’ purchase-related decision-making and purchasing behavior (Aaker and Keller, 1990; Lin et al., 2021). Therefore, the following hypotheses are proposed:

*Hypothesis 1*: Congruence of a virtual anime endorser-product and endorser celebrity (CEPEC) has a positive influence on consumers’ consistency brand attitude (CBA).

*Hypothesis 2*: Congruence of a virtual anime endorser-product and endorser celebrity (CEPEC) has a positive influence on consumers’ consistency purchase intention (CPI).

### Credibility effect of virtual anime endorser–product incongruence and consumer attitudes

Peripheral route constitutes a potential persuasion strategy for low elaboration likelihood and weak arguments (Petty and Cacioppo, 1986). Consumers adopting the peripheral route may form attitudes through a simple linkage of persuasion factors, such as attractive endorsers. The conditions under which moderate incongruence generates more attractive outcomes than congruence is an interesting topic. The moderate incongruence is defined as a moderate level, which is between high and low endorser-produce incongruence and is a peripheral route (Desarbo and Harshamn, 1985). Liang and Lin (2018) investigated the incongruence between a celebrity endorser and a sport product and determined that this incongruence positively influenced consumers’ attitude toward a brand and purchase intention. Therefore, consumers may perceive many situations as “interesting and attractive” because of the curiosity induced by a moderate degree of suddenness, which results in the enhancement of consumers’ attention brand attitudes and purchase intention (Lee and Thorson, 2008; Tang et al., 2012). The positive consequences of moderately incongruent matching have been discussed in consumer psychology literature (e.g., Germelmann et al., 2020). On the basis of the aforementioned discussion, the following hypotheses are proposed:

*Hypothesis 3*: Moderate incongruence between a virtual anime endorser and a product has a positive influence on consumers’ inconsistency brand attitude (IBA).

*Hypothesis 4*: Moderate incongruence between a virtual anime endorser and a product has a positive influence on consumers’ inconsistency purchase intention (IPI).

### Moderating role of product involvement

Involvement is defined as a perception of consumers’ interest, attention, and personal relevance induced by a given situation (Skallerud et al., 2021). With regard to products, involvement affects whether consumers are more active or passive before, during, and after the moment of consumption. Consumers who choose the central route with a high level of product involvement exhibit strong self-motivation to seek and compare other relevant product information before purchasing a product (Liang and Lin, 2018). Less involved consumers utilize the peripheral route to process product messages and focus on convincing clues in certain contexts, such as the product endorser. For individuals with low involvement, an endorser with similarities to the consumers can produce high levels of advertising effectiveness (Albert et al., 2017).

When consumers have a high degree of involvement with products, they tend to adopt a central approach to process information and make decisions after careful consideration based on the content of product information. Therefore, consumers may alter their beliefs and attitudes because of changes in thought, which eventually lead to changes in consumers’ purchase behavior (Skallerud et al., 2021) and brand attitude (Leclerc et al., 1994). By contrast, when consumer have a low degree of involvement, decisions tend to be based on the peripheral information and external clues for a product. Therefore, when the perception of low product involvement changes, changes in purchase intention and brand attitude follow. Prior research reported that product involvement has a major moderating effect on the relationship between consumers’ perceived value and behavior (Gong, 2021). On the basis of the findings of the aforementioned studies, this study speculates that the higher involvement a consumer has toward a product that exhibits congruence or moderate incongruence with its virtual endorser, the higher is the consumer’s attitude toward the brand and purchase intention. This study analyzed the moderating effect of product involvement through the following hypotheses:

*Hypothesis 5a*: Product involvement moderates the relationships among endorser–product congruence, the virtual anime endorser, and attitude toward a brand such that the linkages are stronger when product involvement is higher.

*Hypothesis 5b*: Product involvement moderates the relationships among moderate endorser–product incongruence, the virtual anime endorser, and attitude toward a brand such that the linkages are stronger when product involvement is higher.

*Hypothesis 6a*: Product involvement moderates the relationships among endorser–product congruence, endorser credibility, and purchase intention such that the linkages are stronger when product involvement is higher.

*Hypothesis 6b*: Product involvement moderates the relationships among moderate endorser–product incongruence, the virtual endorser, and purchase intention such that the linkages are stronger when product involvement is higher.

## Materials and methods

### Pretest

This study mainly examined the influence of the congruence between a virtual anime endorser and a product on consumers’ attitude toward the brand and purchase intention. This study first distinguished virtual anime products and general products. The researchers used the pretest questionnaire method to select five well-known anime characters, 10 game products, and 10 general products. A total of 100 participants who were college students in Taiwan were invited at random to evaluate the level of congruence between a virtual anime endorser and the selected products by using a 7-point semantic differential scale ranging from 1 for *strongly disagree* to 7 for *strongly agree*. On the basis of the study of Rifon et al. (2004), the current study employed a three-item scale for congruence. This scale comprised the following items: not compatible/compatible, not a good fit/a good fit, and not congruent/congruent. In addition, participants were asked to rate credibility by using the nine-item scale of Ohanian (1991). Finally, two combinations (i.e., one virtual anime endorser and two product types) were presented to the participants to evaluate their congruence. The formal questionnaire for the main test was designed based on the information obtained in the pretest. According to the results of the experiments conducted with 100 consumers of animation and animation products, Hatsune Miku (33%) created by Yamaha company was selected as the virtual anime endorser for the “Mobile Games - Girl Brand” product. Moreover, “Instruments - an electric piano” was selected as this character’s endorsed product (32%) in the pretest (Table 1). The pretest results identified Hatsune Miku as the most credible figure and congruent source within Mobile Games - Girl Brand, whereas Hatsune Miku was also rated as the least credible and most incongruent source with Instruments - an electric piano. Detailed results of the pretest are indicated in Table 1.

**Table 1:** Pretest results.

	Virtual amine endorser-product congruence				Endorser credibility			
	Mobile Games – Girl Brand		Instrument – an electric piano		Mobile Games – Girl Brand		Instrument – an electric piano	
	*M*	*SD*	*M*	*SD*	*M*	*SD*	*M*	*SD*
Virtual amine celebrity (Hatsune Miku)	6.23	0.95	2.95	1.46	5.43	1.14	3.28	1.22

M = average of 7-point scale items; SD, Standard deviation.

### Formal experiment

In this study, Hatsune Miku was the most popular anime endorser in the pretests. This character was paired with two product types (virtual game products and nonvirtual general products) in the formal questionnaire. The questionnaires of this study contained two endorser–product congruence levels: The Mobile Games - Girl Brand endorsed by Hatsune Miku in the framework of endorser–product congruence and Instrument – an electric piano endorsed by Hatsune Miku in the framework of endorser–product incongruence. The questionnaires of this study were divided into paper questionnaires and online e-questionnaires. Participants were randomly selected from crowds at animation-related events and in animation product stores in Taiwan. A total of 1,002 questionnaires were distributed to participants in the experiment, who accepted them voluntarily and were asked to complete all survey questionnaires. This study distributed 602 questionnaires and presented participants with a small gift (approximately US$2). A total of 400 e-questionnaires were distributed to animation members by LINE in the association. After the exclusion of 83 incomplete questionnaires (8%) from e-questionnaires, a total of 919 valid questionnaires were included in the analysis (92%).

### Measures

#### Endorser–product congruence and incongruence

The scale used for measuring the congruence between the image of the endorser and the product was based on the scale of Lee and Thorson (2008). A 7-point semantic differential scale ranging from 1 for *strongly disagree* to 7 for *strongly agree* was used to assess the following three items: compatible/not compatible, a good fit/not a good fit, and congruent/not congruent. For this study, the Cronbach’s alpha (*α*) was 0.81.

#### Endorser credibility

This scale was developed by Ohanian (1991) to measure respondents’ assessment of the effectiveness of purchased products on a 7-point scale ranging from 1 for *strongly disagree* to 7 for *strongly agree*. The participants were asked to evaluate the following items: attractive/unattractive, classy/not classy, beautiful/ugly, elegant/plain, sexy/not sexy, dependable/undependable, honest/dishonest, reliable/unreliable, sincere/insincere, trustworthy/untrustworthy, expert/not an expert, experienced/inexperienced, knowledgeable/unknowledgeable, qualified/unqualified, and skilled/unskilled (*α* = 0.92). Many studies have empirically validated the aforementioned scale (e.g., Lee and Koo, 2016).

#### Product involvement

This scale developed by Zaichkowsky (1985) was used to examine product involvement on a four-item (e.g., unimportant/important) with 7-point semantic differential scale ranging from 1 for *strongly disagree* to 7 for *strongly agree*. The higher the obtained score, the higher was the product involvement (i.e., The Mobile Games - Girl Brand and Instrument – an electric piano). The Cronbach’s alpha (*α*) of this study was 0.95.

#### Attitude toward the brand

According to the research of Leclerc et al. (1994) on brand attitudes, three items were used to measure the degree of consumer attention to brand attitudes on a 7-point Likert scale ranging from 1 for *strongly disagree* to 7 for *strongly agree*. The higher the obtained score, the higher was the emphasis on the attitude toward the brand. For this study, the Cronbach’s alpha (*α*) was 0.96.

#### Purchase intention

The scale developed by Yi (1990) was used to examine purchase intention. A 7-point semantic differential scale ranging from 1 for *strongly disagree* to 7 for *strongly agree* was used to rate the following items: very likely/very unlikely, probable/improbable, and possible/impossible. The Cronbach’s alpha (*α*) of this study was 0.92.

#### Control variables

This study controlled for sex, age, marriage status, income, education, and employment status to reduce any potential influence of demographic characteristics on the results (Turnley and Feldman, 1999). Problems in achieving generalizability can be reduced by controlling for demographic variables (Tomprou et al., 2012). Age was assessed using open-ended items.

## Results

### Manipulation check

This study conducted one-way ANOVA to analyze whether the two operationalized levels of virtual anime endorser–product congruence differed significantly from each other. The average ratings for virtual anime endorser–product congruence (i.e., Mobile Games - Girl Brand) and virtual anime endorser–product incongruence (i.e., Instrument - an electric piano) were 6.23 and 2.95, respectively. The difference between high and low congruence was significant (*F* = 3.64, *p* < 0.01). The average value of endorser credibility (i.e., Hatsune Miku) was 5.43.

### Main effects

The correlation coefficients presented in Table 2 indicate the positive correlations between congruence of endorser-product and endorser celebrity (CEPEC) and consistency brand attitude (CBA), which is H1 (*r* = 0.41, *p* < 0.05) and incongruence of endorser-product and endorser celebrity (IEPEC) and inconsistency brand attitude (IBA), which is H3 (*r* = 0.11, *p* < 0.01). CEPEC significantly influenced consistency purchase intention (CPI), which is H2 (*r* = 0.47, *p* < 0.01), whereas IEPEC had a nonsignificant effect on inconsistency purchase intention (IPI), which is H4 (*r* = 0.09, *p* < 0.05).

**Table 2:** Means, standard deviations, Cronbach’s alpha, and intercorrelations among study.

Variables	*M*	*SD*	1	2	3	4	5	6	7	8	9	10	11	12	13	14
1. Sex	1.41	0.49	(−)													
2. Age	2.13	0.95	−0.25^**^	(−)												
3. Marriage	1.98	0.25	0.01	−0.27^**^	(−)											
4. Income	1,49	0.82	−0.29^**^	0.55^**^	−0.23^**^	(−)										
5. Education	2.21	1.04	−0.11^**^	0.55^**^	−0.52^**^	−0.09^**^	(−)									
6. Vocation	5.71	2.05	0.25^**^	−0.11^**^	0.10^**^	−0.08^*^	−0.09^**^	(−)								
7. CEPEC	5.43	1.46	0.09^**^	0.11^**^	−0.02	−0.02	−0.08^*^	0.01	(0.81)							
8. IEPEC	2.96	1.14	−0.03	−0.01	−0.03	−0.11^**^	−0.02	−0.04	0.11^**^	(0.81)						
9. EC	6.23	0.95	−0.05	−0.13^*^	−0.07^*^	−0.11^**^	−0.12^**^	−0.03	0.28^**^	0.16^**^	(0.92)					
10. PI	5.10	1.31	0.04	−0.07^*^	−0.06	−0.12^**^	−0.11^**^	−0.01	0.55^**^	0.09^**^	0.36	(0.95)				
11. CPI	5.31	1.40	0.08^*^	−0.04	−0.06	−0.11^**^	−0.12^**^	−0.01	0.47^**^	0.08^*^	0.42^**^	0.76^**^	(0.92)			
12. ICPI	5.23	1.41	0.05	−0.02	0.04	0.06	−0.06	−0.02	0.20^**^	0.09^*^	0.29^**^	0.42^**^	0.48^**^	(0.92)		
13. CBA	5.66	1.14	−0.04	−0.04	−0.02	−0.12^**^	−0.12^**^	−0.02	0.41^**^	0.13^**^	0.55^**^	0.62^**^	0.66^**^	0.40^**^	(0.96)	
14. IBA	5.79	1.10	−0.03	−0.05	−0.08^*^	0.03	−0.07^*^	−0.04	0.17^**^	0.11^**^	0.43^**^	0.35^**^	0.39^**^	0.62^**^	0.50^**^	(0.96)

Coefficient alphas are in parentheses on the diagonal. CEPEC, Congruence of endorser-product and endorser celebrity; IEPEC, Incongruence of endorser-product and endorser celebrity; EC, Endorser credibility; PI, Product involvement; CPI, Consistency purchase intention; ICPI, Inconsistency purchase intention; CBA, Consistency brand attitude; IBA, Inconsistency brand attitude.

* *p* < 0.05 and

** *p* < 0.01.

### Multivariate analysis of variance

A multivariate analysis of variance (MANOVA) was conducted with two independent variables, namely the virtual anime celebrity and virtual anime endorser–product congruence, and two dependent variables, namely attitude toward the brand and purchase intention. The two-way MANOVA interaction between the virtual endorser and virtual endorser–product congruence was significant [Wilks’ lambda (*λ*) = 0.47; *F* = 2.99, *p* < 0.01], which allowed ANOVAs to be performed for the two dependent variables with the protection of the Type 1 error rate. MANOVA was also performed using the virtual anime endorser and virtual endorser–product incongruence as independent variables and attitude toward the brand and purchase intention as dependent variables. The results of the two-way MANOVA interactions between the virtual endorser and virtual endorser–product incongruence were significant [Wilks’ lambda (*λ*) = 0.53; *F* = 3.16, *p* < 0.01], which allowed ANOVAs to be performed for the two dependent variables with the protection of the Type 1 error rate.

The results indicated that the congruence between a virtual anime endorser and a product significantly influenced attitude toward the brand [*F*(1, 117) = 3.22, *p* < 0.01]; thus, Hypothesis 1 is supported. The congruence between a virtual anime endorser and a product significantly influenced purchase intention [*F*(1, 117) = 2.58, *p* < 0.01]; thus, Hypothesis 2 is supported. Moreover, the interaction of virtual endorser–product incongruence with the endorser significantly influenced attitude toward the brand [*F*(1, 95) = 4.11, *p* < 0.01]; thus, Hypothesis 3 is supported. Finally, the interaction of virtual endorser–product incongruence with the endorser significantly influenced purchase intention [*F*(1, 95) = 2.77, *p* < 0.01]; thus, Hypothesis 4 is supported.

### Regression analysis

The regression analysis verified Hypotheses 1–6b in this study. The control variables were input in the first step, the main effects were input in the second and third steps, and the moderating variable was input in the final step. The total effect and magnitude of the results in this study demonstrated the significant effects of the interaction in the moderation model. The following text indicates the key findings regarding the effects of endorser–product congruence and endorser–product incongruence on attitude toward the brand and purchase intention in the regression analysis.

First, as presented in Table 3, the results of the hierarchical multiple regression analyses indicate that virtual anime endorser–product congruence had a positive influence on attitude toward the brand [*β* = 0.11, *p* < 0.01]; thus, Hypothesis 1 is supported. The results of step 4 listed in Table 3 indicate that product involvement moderated the relationship between virtual anime endorser–product congruence and attitude toward the brand (*β* = 0.37, *p* < 0.01; *F* = 3.68, *p* < 0.05); thus, Hypothesis 5a is supported. In addition, this study employed PROCESS macro for SPSS 22.0 with 10,000 bootstrap samples to examine H5a. Table 4 shows that the effect of virtual anime endorser–product congruence on consistency brand attitude whether the degree of product involvement was high or low. Particularly, the direct effect is strengthened when the consumers have higher product involvement (low = 1.10, mean = 0.21, and high = 0.58). As presented in Table 5, the analysis results also support Hypothesis 2, which states that virtual anime endorser–product congruence has a positive influence on consumers’ purchase intention (*β* = 0.19, *p* < 0.01). The results of step 4 presented in Table 5 indicate that product involvement moderated the relationship between virtual anime endorser–product congruence and purchase intention (*β* = 0.52, *F* = 7.41, *p* < 0.01); thus, Hypothesis 6a is supported. In addition, this study employed PROCESS macro for SPSS 22.0 with 10,000 bootstrap samples to examine H6a. Table 6 shows that the effect of virtual anime endorser–product congruence on consistency purchase intention whether the degree of product involvement was high or low. Particularly, the direct effect is strengthened when the consumers have higher product involvement (low = 0.91, mean = 0.08, and high = 0.79).

**Table 3:** Results of product involvement as a moderating role between virtual anime endorser-product congruence and attitude toward the brand.

Consistency brand attitude (*β*)				
Predictors	Step 1	Step 2	Step 3	Step 4
Gender	−0.02	−0.02	−0.02	−0.01
Age	0.10	0.11^*^	0.11^**^	0.12^*^
Marriage	−0.03	−0.02	−0.02	−0.02
Salary	−0.11^**^	−0.11^**^	−0.11^*^	−0.11^*^
Education	−0.04	−0.04	−0.04	−0.04
Vocation	−0.05	−0.05	−0.05	−0.05
Product involvement		0.25^**^	0.22^**^	0.21^**^
Interaction of CEPEC			0.11^**^	0.09^*^
CEPEC * Product involvement				0.37^**^
*R^2^*	0.01	0.02	0.02	0.03
Adjusted *R^2^*	0.01	0.01	0.01	0.02
*F*	2.12^*^	0.74	7.30^**^	3.68^*^

CEPEC, Congruence of endorser-product and endorser celebrity.

* *p* < 0.05 and

** *p* < 0.01.

**Table 4:** Results for the effect of CPEC on consistency brand attitude across levels of product involvement.

Moderator variable	Effect size	*SE*	LL 95% CI	UL 95% CI
Low product involvement	1.10	0.43	0.26	1.94
Middle product involvement	0.21	0.07	0.07	0.34
High product involvement	0.58	0.10	0.38	0.77
Index	0.16	0.06	0.03	0.31

Bootstrap samples = 10,000. SE, Standard error; LL, Lower limit; UL, Upper limit; CI, Confidence interval; CPEC, Congruence of endorser-product and endorser celebrity.

**Table 5:** Results of product involvement as a moderating role between virtual anime endorser-product congruence and purchase intention.

Consistency purchase intention (*β*)				
Predictors	Step 1	Step 2	Step 3	Step 4
Gender	−0.04	−0.04	−0.05	−0.03
Age	0.05	0.06	0.06	0.07
Marriage	−0.01	−0.01	−0.01	−0.00
Salary	−0.10^*^	−0.10^*^	−0.10^*^	−0.10^*^
Education	−0.04	−0.04	−0.04	−0.04
Vocation	−0.07	−0.06	−0.06	−0.06
Product Involvement		0.26^**^	0.23^**^	0.22^**^
Interaction of CEPEC			0.19^**^	0.14^**^
CEPEC * Product Involvement				0.52^**^
*R^2^*	0.01	0.02	0.02	0.03
Adjusted *R^2^*	0.01	0.01	0.01	0.02
*F*	1.97^*^	2.29	4.08^*^	7.41^**^

CPEC, Congruence of endorser-product and endorser celebrity.

* *p* < 0.05 and

** *p* < 0.01.

**Table 6:** Results for the effect of CPEC on consistency purchase intention across levels of product involvement.

Moderator variable	Effect size	*SE*	LL 95% CI	UL 95% CI
Low product involvement	0.91	0.37	0.19	1.13
Middle product involvement	0.08	0.07	0.05	0.37
High product involvement	0.79	0.08	0.63	0.93
Index	0.04	0.05	0.06	0.21
**Moderator variable**	**Effect size**	***SE***	**LL 95% CI**	**UL 95% CI**
Low product involvement	0.59	0.13	0.32	0.85
Middle product involvement	0.04	0.04	0.04	0.26
High product involvement	0.68	0.04	0.60	0.77
Index	0.03	0.04	0.03	0.26

Bootstrap samples = 10,000. SE, Standard error; LL, Lower limit; UL, Upper limit; CI, Confidence interval; CPEC, Congruence of endorser-product and endorser celebrity.

Second, as presented in Table 7, moderate virtual anime endorser–product incongruence had a positive influence on attitude toward the brand (*β* = 0.19, *p* < 0.01); thus, Hypothesis 3 is supported. The results of step 4 presented in Table 7 indicate that product involvement moderated the relationship between virtual anime endorser–product incongruence and attitude toward the brand (*β* = 0.61, *F* = 15.39, *p* < 0.01); thus, Hypothesis 5b is supported. In addition, this study employed PROCESS macro for SPSS 22.0 with 10,000 bootstrap samples to examine H5b. Table 8 shows that the effect of virtual anime endorser–product incongruence on inconsistency brand attitude whether the degree of product involvement was high or low. Particularly, the direct effect is strengthened when the consumers have higher product involvement (low = 0.80, mean = 0.26, and high = 0.82). As presented in Table 9, the results of this study also support Hypothesis 4, which states that moderate virtual anime endorser–product incongruence has a positive influence on consumers’ purchase intention (*β* = 0.21, *p* < 0.01). The results of step 4 presented in Table 9 reveal that product involvement moderated the relationship between virtual anime endorser–product incongruence and purchase intention (*β* = 0.65, *F* = 18.38, *p* < 0.01); thus, Hypothesis 6b is supported. In addition, this study employed PROCESS macro for SPSS 22.0 with 10,000 bootstrap samples to examine H6b. Table 10 shows that the effect of virtual anime endorser–product incongruence on inconsistency purchase intention whether the degree of product involvement was high or low. Particularly, the direct effect is strengthened when the consumers have higher product involvement (low = 0.59, mean = 0.04, and high = 0.68).

**Table 7:** Results of product involvement as a moderating role between virtual anime endorser-product incongruence and attitude toward the brand.

Inconsistency brand attitude (*β*)				
Predictors	Step 1	Step 2	Step 3	Step 4
Gender	−0.03	−0.03	−0.03	−0.02
Age	−0.14^*^	−0.07	−0.06	−0.05
Marriage	−0.10^**^	−0.07^*^	−0.07^*^	−0.07^*^
Salary	0.06	0.06	0.07	0.06
Education	−0.03	−0.02	−0.02	−0.02
Vocation	−0.07	−0.03	−0.02	−0.03
Product Involvement		0.34^**^	0.23^**^	−0.11
Interaction of IEPEC			0.19^**^	0.24^**^
IEPEC * Product Involvement				0.61^**^
*R^2^*	0.02	0.13	0.15	0.17
Adjusted *R^2^*	0.02	0.12	0.15	0.16
*F*	3.40^**^	113.83^**^	23.81^**^	15.39^**^

IEPEC, Incongruence of endorser-product and endorser celebrity.

* *p* < 0.05 and

** *p* < 0.01.

**Table 8:** Results for the effect of IEPEC on inconsistency brand attitude across levels of product involvement.

Moderator variable	Effect size	*SE*	LL 95% CI	UL 95% CI
Low product involvement	0.80	0.18	0.46	1.13
Middle product involvement	0.26	0.06	0.15	0.37
High product involvement	0.82	0.06	0.71	0.93
Index	0.56	0.08	0.07	0.19

Bootstrap samples = 10,000. SE, Standard error; LL, Lower limit; UL, Upper limit; CI, Confidence interval; IEPEC, Incongruence of endorser-product and endorser celebrity.

**Table 9:** Results of product involvement as a moderating role between virtual anime endorser-product incongruence and purchase intention.

Inconsistency purchase intention (*β*)				
Predictors	Step 1	Step 2	Step 3	Step 4
Gender	−0.03	−0.03	−0.03	−0.02
Age	−0.14^*^	−0.07	−0.06	−0.05
Marriage	−0.10^**^	−0.07^*^	−0.07^*^	−0.06^*^
Salary	0.07	0.07	0.08	0.07
Education	−0.03	−0.02	−0.02	−0.02
Vocation	−0.07	−0.03	−0.03	−0.03
Product involvement		0.36^**^	0.25^**^	0.20^**^
Interaction of IEPEC			0.21^**^	0.19^**^
IEPEC * Product Involvement				0.65^**^
*R^2^*	0.02	0.14	0.16	0.18
Adjusted *R^2^*	0.02	0.13	0.16	0.17
*F*	4.30^**^	121.69^**^	26.81^**^	18.38^**^

IEPEC, Incongruence of endorser-product and endorser celebrity.

* *p* < 0.05 and

** *p* < 0.01.

**Table 10:** Results for the effect of IEPEC on inconsistency purchase intention across levels of product involvement.

Moderator variable	Effect size	SE	LL 95% CI	UL 95% CI
Low product involvement	0.59	0.13	0.32	0.85
Middle product involvement	0.04	0.04	0.04	0.26
High product involvement	0.04	0.04	0.60	0.77
Index	0.03	0.04	0.03	0.26

Bootstrap samples = 10,000. SE, Standard error; LL, Lower limit; UL, Upper limit; CI, Confidence interval; IEPEC, Incongruence of endorser-product and endorser celebrity.

## Discussion

This study examined that virtual anime endorser–product congruence and moderate virtual anime endorser–product incongruence influence consumers’ attitude toward a brand and purchase intention by using a questionnaire that matched a virtual anime endorser with an anime-related product and a general product. In addition, this study investigated that product involvement moderates the relationships between the virtual anime endorser–product congruence/moderate anime endorser–product incongruence and consumers’ behaviors (i.e., brand attitude and purchase intention). In particular, the effects of virtual endorser credibility on consumers’ attitudes were more significant under high product involvement than under low product involvement. The findings of this study indicate that moderate virtual anime endorser–product incongruence plays a significant role in increasing consumers’ purchase behaviors. Consumers with high product involvement tend to exhibit purchase intention when the endorser endorses incongruent products.

The results of this study revealed that the participants with low product involvement had a high level of brand attitude when the endorser endorsed incongruent products. The results of this study indicate that highly involved consumers are affected by peripheral clues under endorser–product incongruence. After introduction to the product through the virtual endorser, the participants of this study exhibited high brand attitude. The findings of previous studies suggest that the same clues may simultaneously induce information processing through the central and peripheral paths (Petty and Cacioppo, 1986; Tang et al., 2012; Lee and Koo, 2016). The virtual anime endorser is a peripheral clue when endorsing physical products and a central clue when endorsing virtual products (Petty et al., 1987). Virtual anime endorsers may be suitable for endorsements of anime-related products and physical products, especially inconsistent endorsements. Consumers with high involvement may be affected by peripheral clues, such as the anime virtual endorser. For consumers with a high degree of product involvement, endorser–product congruence may be regarded as a central clue in the formation of brand attitude. The aforementioned results have rarely been obtained in studies on virtual anime endorser–product congruence.

### Theoretical implications

This study has several critical theoretical implications and enhances the depth of understanding of endorsement studies in consumer psychology. First, previous studies on endorsers and consumers have mainly focused on celebrity endorsers (e.g., Liang and Lin, 2018). Little research has been conducted on the effects of virtual endorser–product incongruence on consumer choice. This study demonstrated that consumers have different brand attitudes toward products under different levels of consistency between endorsers and products and emphasized the role of product involvement as a moderating factor in the relationships of virtual endorser–product congruence and moderate virtual endorser–product incongruence with brand attitudes.

Second, no research has focused on the endorsement of physical products by virtual anime characters. Most studies that have referred to the ELM considered products to be only single peripheral clues. Therefore, peripheral clues have been considered to affect only low-level product involvement. However, this study verified that when consistency between an endorser and a product is considered irrelevant, the attitude of high-involvement consumers may be determined by the quality and value of the product. Conversely, the attitude of those with low involvement may be determined by the virtual anime endorser. Therefore, this study investigated the effect of the degree of product involvement on the relationship between virtual anime endorser–product incongruence and brand attitudes.

Third, the results of this study are consistent with those of Lee and Koo (2016), who suggested that future research include product involvement as a moderator in the analysis. This study examined the effects of endorser–product congruence and moderate endorser–product incongruence for different product categories and demonstrated that the level of product involvement has critical moderating influences on the effects of endorser–product congruence, moderate endorser–product incongruence, and endorser credibility on consumer behaviors.

### Practical implications

This study analyzed whether product involvement strengthened the relationships of virtual anime endorser–product congruence and virtual anime endorser–product incongruence with consumer behaviors. This study suggests companies with limited endorsement budgets on how to identify the ideal endorser. For consumers with low or high product involvement, companies can select endorsers who exhibit moderate incongruence with their product but must be cautious when choosing highly attractive endorsers. Highly attractive endorsers mostly serve to attract consumers’ attention to the product rather than to elevate the brand attitude of the product. Virtual endorsers are therefore advantageous because their attractiveness can draw the attention and curiosity of consumers, which can stimulate product purchase (Hsu et al., 2018). Companies have employed virtual anime characters to endorse products on certain occasions despite the incongruence of the brand image or product with the virtual character’s image.

Managers can draw on virtual characters’ attractiveness to increase consumer purchase intention and product recognition. This approach is effective for virtual consumers who are already familiar with virtual characters. These consumers may already have a high psychological attachment to the virtual anime endorsers at the time when they are presented with endorser–product incongruence. Even if the product involvement of consumers decreases, the effects of endorser credibility on general consumers with a fascination for anime characters can be maintained. These consumers may require strong image congruence between the endorser and the product for the endorser credibility to take full effect. Thus, marketing companies may use the effects of virtual anime endorser–product congruence and moderate virtual anime endorser–product incongruence to increase consumer’s purchase intention and brand attitude.

### Limitations and future research

This study has some limitations. First, although the questionnaire was administered at animation-related event venues, such as animation and accessories stores, this study may not confirm whether all the formal participants were familiar with animation-related endorsers or whether animation-related endorsers could serve as ideal virtual endorsers for the participants. However, Hatsune Miku was the virtual anime endorser in this study, and the results indicated that most animation consumers could accept Hatsune’s role as a product endorser. Other virtual anime endorser characters may be included in future research to provide additional information.

Second, this study examined the relationships of endorser–product congruence and moderate endorser–product incongruence with consumers’ attitudes (i.e., brand attitude and purchase intention). Research has indicated that the attractiveness of the endorser may affect consumer intent and brand attitude (Liu et al., 2007). In this study, the attractiveness of Hatsune Miku may have influenced the effectiveness of endorsement advertising. Therefore, future research may include the construct of attractiveness to examine its effect on the relationships of endorser–product congruence and moderate endorser–product incongruence with brand attitude and purchasing intention.

Third, the sample of this study was relatively small, as it is characteristic of single-source data from employees, which may raise concerns about common method bias. In addition, the results of this study may not be generalized to all consumers in other countries even if the findings of this study found that the influences of virtual anime endorser–product congruence and moderate the endorser–product incongruence on consumer brand attitude and purchase intention. Future research investigating whether these prototypes differ in other countries would be valuable and may examine diverse cultural samples to reduce generalizability issues.

## Conclusion

This study validated that virtual endorser-product congruence and moderate virtual endorser-product incongruence affect consumers’ attitude toward a brand and purchase intention. In addition, product involvement was identified as a critical moderator in the relationships of virtual anime endorser–product congruence and moderate virtual anime endorser–product incongruence with consumer behaviors. Managers cannot maximize the effectiveness of celebrity endorsers if they consider only one of the two key factors. Furthermore, virtual anime endorser–product congruence and incongruence significantly influence the relationship between endorser credibility and consumer responses. Therefore, the use of virtual anime characters as endorsers can be beneficial for enhancing consumers’ attitude toward a brand and purchase intention.

## Data availability statement

The original contributions presented in the study are included in the article/supplementary material, further inquiries can be directed to the corresponding author.

## Ethics statement

Ethical review and approval was not required for the study on human participants in accordance with the local legislation and institutional requirements. The patients/participants provided their written informed consent to participate in this study.

## Author contributions

All authors confirm being the contributors of this work and approved it for publication.

## Conflict of interest

The authors declare that the research was conducted in the absence of any commercial or financial relationships that could be construed as a potential conflict of interest.

## Publisher’s note

All claims expressed in this article are solely those of the authors and do not necessarily represent those of their affiliated organizations, or those of the publisher, the editors and the reviewers. Any product that may be evaluated in this article, or claim that may be made by its manufacturer, is not guaranteed or endorsed by the publisher.
